# Distributed Architecture to Integrate Sensor Information: Object Recognition for Smart Cities

**DOI:** 10.3390/s20010112

**Published:** 2019-12-23

**Authors:** Jose-Luis Poza-Lujan, Juan-Luis Posadas-Yagüe, José-Enrique Simó-Ten, Francisco Blanes

**Affiliations:** University Institute of Control Systems and Industrial Computing (ai2), Universitat Politècnica de València (UPV) Camino de Vera, s/n, 46022 Valencia, Spain; jposadas@ai2.upv.es (J.-L.P.-Y.); jsimo@ai2.upv.es (J.-E.S.-T.); fblanes@ai2.upv.es (F.B.)

**Keywords:** smart environment, smart sensors, distributed architectures, object detection, information integration, smart cities

## Abstract

Object recognition, which can be used in processes such as reconstruction of the environment map or the intelligent navigation of vehicles, is a necessary task in smart city environments. In this paper, we propose an architecture that integrates heterogeneously distributed information to recognize objects in intelligent environments. The architecture is based on the IoT/Industry 4.0 model to interconnect the devices, which are called smart resources. Smart resources can process local sensor data and offer information to other devices as a service. These other devices can be located in the same operating range (the edge), in the same intranet (the fog), or on the Internet (the cloud). Smart resources must have an intelligent layer in order to be able to process the information. A system with two smart resources equipped with different image sensors is implemented to validate the architecture. Our experiments show that the integration of information increases the certainty in the recognition of objects by 2–4%. Consequently, in intelligent environments, it seems appropriate to provide the devices with not only intelligence, but also capabilities to collaborate closely with other devices.

## 1. Introduction

The growth of cities has given rise to an environment populated by increasingly intelligent and more connected devices, practically all of which have sensors and actuators featuring very different capabilities. Each one of these devices can be considered as a control node; however, as the devices are interconnected and can communicate to share their resources, the concept of a control node can be reconsidered as the concept of an intelligent resource that provides services to the rest of the devices [1].

In addition, heterogeneous devices provide complementary information, which can be used to enrich the overall knowledge. Consequently, a distributed system in which the devices are intelligent can be defined as an intelligent system or smart system [2]. Smart systems perform their tasks in dynamic environments with multiple features and changing conditions; for example, urban environments are dynamic, unpredictable systems, that provide challenges for the application of intelligent systems.

Therefore, continuous and accurate knowledge of the environment is necessary to provide autonomy and interaction. Subsystems, such as robots or vehicle navigation systems, need to know the environment to perform their tasks, such as the planning of trajectories or the execution of missions [3].

Object recognition is one of the typical functionalities required by the elements of a smart city. For example, vehicles need to recognise traffic signals for autonomous driving [4]. Other applications of the detection of objects in cities are the detection of people [5] or the detection of vehicles [6], generally to improve road safety or the comfort of citizens.

The diversity of available sensors is especially relevant in smart cities. In the same street, it is possible to find traffic cameras at fixed points, as well as navigation cameras in vehicles. Both types of cameras can co-operate in the identification of objects on the road. In this way, it is possible to access a system which allows for distinguishing between authorised objects (such as cleaning and work signals) and non-authorised objects (such as garbage or even potentially dangerous objects).

**Figure 1:**
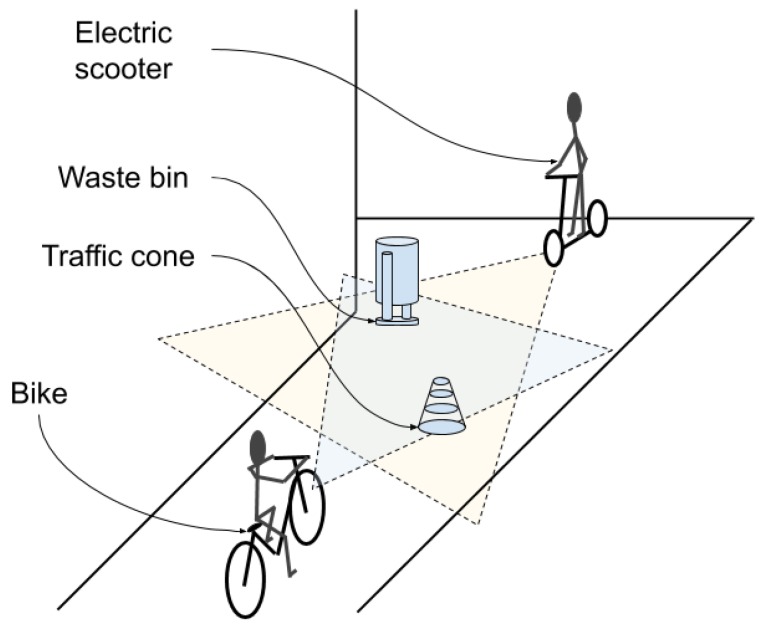
Urban environment in which various smart devices can collaborate in order to detect objects more accurately.

Figure 1 shows an example in which various devices detect objects in an urban environment. If both vehicles have cameras, they are able to detect objects based on the patterns they have and the type of camera. When the vehicles are close to one another in the environment, they are able to communicate in order to increase the certainty of their observations. In this way, if the driver of the bike is interested in looking for a waste bin and the electric scooter has recognised the object with more certainty, the electric scooter will be able to facilitate the location of the waste bin for the driver of the bike. All the previous tasks must be done in a time range appropriate to each situation; for example, to not miss the trash or to avoid the traffic cone. The other vehicle can use information integration to be sure that the object detected is a traffic cone, to know if the cone can be avoided at the current speed, or if they must decrease their speed to avoid it.

At present, the elements of smart cities are increasing their capacity for processing and communication. Therefore, it is interesting to study how the efficiency of such systems can be improved when their heterogeneous elements collaborate, in order to integrate the information they have collected.

The aim of this paper is to present the study and implementation of a latency-aware solution to integrate sensory information, in order to increase certainty in the object recognition process.

Therefore, we propose an architecture that provides the necessary communications system, which is easily accessed and allows for the integration of information from all types of distributed devices which can be found in a smart city. These devices may be physical, such as an ultrasonic sensor, or logical, such as a process that generates an average value using the temperatures obtained from several physical devices. From the idea of logical devices, the architecture can be structured in different levels, according to the abstraction of the data that the devices use or generate. At the same time, the devices may be physically located according to their mutual proximity, which determines the communication mechanism to be used. In any case, the architecture has to provide sufficient message latency times for the devices to be capable of adequate decision-making.

To test the architecture, a use-case scenario is presented for object recognition. The objective is to increase certainty in the recognition of specific objects by integrating information from different distributed sensors. The idea is to test how the architecture allows for the agile integration of heterogeneous information from various devices to improve the recognition of objects, regardless of the recognition methods used. Better methods may lead to better results, but that is not our goal.

The paper is organised as follows: Once the aim of the investigation has been contextualised, Section 2 presents the related work. Then, Section 3 describes the proposed architecture. Section 4 presents the use-case for object recognition and Section 4.1 and Section 4.2 describe the system implemented with the sensors and methods and the scenario in which the system was tested, respectively. Next, Section 5 presents the results of the experiments performed for the recognition of two different objects. The results obtained verify how the integration of information from the services provided by the smart resources improves the object detection accuracy. Finally, the conclusions are drawn and some of the future lines of research to be developed are presented.

## 2. Related Work

In order to contextualise and focus the present study, this section presents the most-used current paradigms to design architectures that can be applied in smart cities. Next, we focus on how devices can provide intelligence to cities by detecting and recognising objects in the environment and the role of communication between these devices will be discussed. Finally, the context of the present work is located within some architectures of intelligent cities that use the paradigms presented.

Within the field of architecture of cyber-physical systems [7], our work is related to the current technological trend of ubiquitous and decentralised computational paradigms [8]. From the beginning of artificial intelligence (AI) in the 1950s, how to apply AI in cities has always been one of the main concerns. Consequently, architectures to manage intelligence in a city is one of the main research fields. A large number of paradigms that can be used to inspire an architecture for a smart city have been proposed. These include paradigms such as ubiquitous computing [9], Industry 4.0 [10], or Internet of Things (IoT) [11].

**Figure 2:**
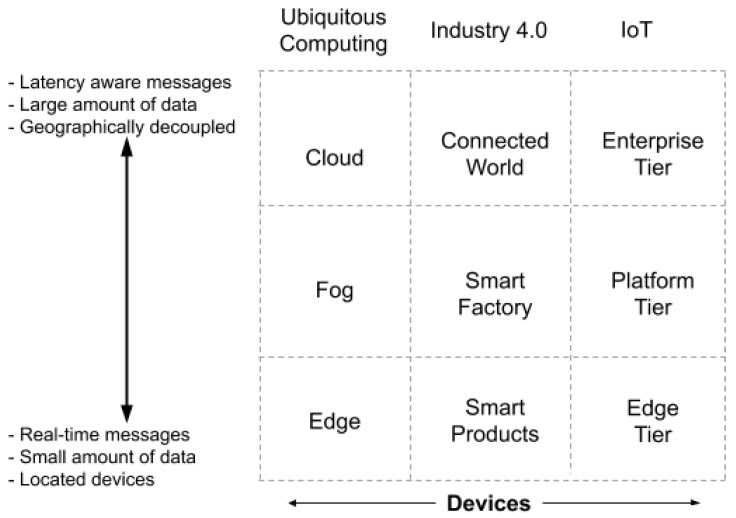
Decentralised computational paradigms.

The fact that the elements of a cyber-physical system can exchange information, decoupling their location in the hierarchy of the architecture, has led to the emergence of these paradigms. These paradigms organise the components in layers according to certain characteristics, such as the geographical scope, amount of data, or message latency [12]. Figure 2 shows the different layers considered by the paradigms. Based on the dimensions proposed in [12], the ubiquitous computing, Industry 4.0, and Internet of Things (IoT) paradigms stand out as very suitable for the design of any system that provides intelligence to a city.

IoT has mainly been applied to urban systems which are based on devices with many sensors. A device with many sensors may be overloaded, or its sensors can be used very sporadically, with a consequent loss of efficiency. To reduce the load on each device, it is important to be able to exchange sensory information between devices. For this, devices have to be able to communicate not only with devices at their own semantic level, but also with any element of the system.

The Reference Architectural Model Industry 4.0 (RAMI 4.0) [13] is based on smart products, which the ubiquitous computing and IoT [14] paradigms place in the edge layer [15]. These devices have a scope of sensorization and action on the order of a few meters, as well as very fast reaction times. Examples of these products range from robots in an assembly line or smart street lamps, placed in the same street, which optimise energy consumption. When several devices at the edge level communicate or interact on a wider level (spatially or temporally), it is called the smart factory or platform tier. The ubiquitous paradigm places these devices, or processes, in a layer called the fog layer [15]; some examples include when monitoring the performance of all robots in a factory or when managing the lighting of a neighbourhood in a city. Finally, when the devices are connected to exchange large amounts of data or when there is a longer-term reaction, it is called a connected world, the business level, or the well-known concept of the cloud layer [9]. The control architectures of smart cities fit perfectly into these models [16].

The addition of micro-controllers and micro-processors to sensor devices increases the information capacity that the sensors can provide. These devices are usually called smart or intelligent sensors, respectively [17]. When the sensor includes some advanced processing and (in some cases) actuators, some authors have called them smart devices [18]. Adding a communication interface allows smart devices to share information and, consequently, increase the knowledge of the environment. The use of smart devices has grown from environments, like smart cities [19], to the concept of smart objects, where these devices have become part of the daily lives of people [20].

Consequently, the present situation is that sensors can send processed information, rather than raw data. The result is that sensor networks can form distributed systems that integrate sensor information in order to take advantage of the processed information [21]. When there are different distributed devices in the network, some interesting problems arise. One of the problems is achieving a balance between the number of devices used and the correct use of their sensors; that is, when a new device is introduced, its sensors should help to increase the probability of success when detecting and recognising an object. Consequently, the composition and connection between the devices will determine how to recognise the objects. For example, two devices with Red, Green, and Blue (RGB) sensors will recognise the same texture with a similar probability. However, the probability of success could increase by using another type of sensor which reinforces the process; for example, a thermal camera which can distinguish between different ink types.

Sensors can help to understand the environment by detecting objects and some of their characteristics. However, when the objects detected have to be classified and recognised, a set of patterns with which to compare [22] is necessary. For example, the shape of a box can be detected by means of a 3D sensor, but the same box can have different textures, so it is also necessary to use other types of sensors (e.g., an RGB sensor) to recognise what type of box it is. Therefore, using heterogeneous sensors to detect and recognise the objects present in an environment can increase the probability of success in recognising the object. When working with heterogeneous sensors, their information must be merged, usually by remotely creating sensor networks [23].

To monitor public spaces in smart cities, there are some functionalities that depend on object detection and recognition. The previously mentioned examples are perfect ecosystems in which to use the fog or edge computation paradigms. The heterogeneity of the devices, places in the edge, and the possibility to process sensor information allow devices, such as streetlights, to make decisions, such as deciding whether or not they should be lit, or whether traffic lights adapt their duration to the length of vehicle queues. The fact that devices are placed in common spaces (e.g., the same street for streetlights or a common neighbourhood for traffic lights) suggests that fog computing allows for the creation of federations of nodes or even clusters dependent on the same field of action.

**Figure 3:**
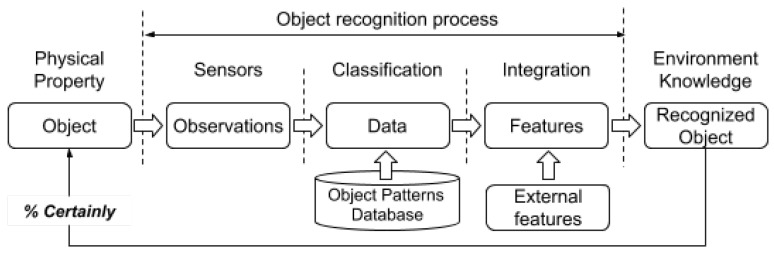
Overview of the components of the object recognition process in the integration of sensory information.

In summary, acquiring characteristics of the environment to associate them with specific objects implies a sequence of actions, as shown in Figure 3.

The inclusion of object detection in the environment map adds a difficulty and forces the use of advanced sensors. Consequently, when there are many sensors, the quality of data fusion is dependent on the fusion mechanism [24]. Once a certain precision in the detection of the object and its characteristics has been achieved, it should be possible to classify the object [25]. The classification of an object requires the use of patterns in order to compare the percentage of similarity [26]. Therefore, in an object recognition system, a database of patterns is necessary.

The different components of the process presented in Figure 3 can be located at any of the levels presented in Figure 2. The sensors belong to the edge, while classification and integration are processes that can occur at the local device (edge), at any nearby device (fog), or in dedicated servers (cloud). As a result, communication middleware is required for different types of devices to co-operate and communicate. This middleware has to allow subscription to specific services, offering a balanced network load. The publish–subscribe [27] paradigm is one of the most suitable, as it allows the physical decoupling of the devices involved in the communication and the connection of each device to the information that it is interested in.

Smart city architectures based on the ubiquitous computing, Industry 4.0, or IoT paradigms need to decide which components have to be placed in the cloud, the fog, or the edge. In [28], a cloud-based architecture has been presented. In this case, some of the data is processed in the cloud-computing infrastructure, while the edge handles the rest. In [29], a multi-layer smart city architecture has been presented. In this case, the sensors communicate with advanced services after the integration phase, which occurs directly in the cloud. Of the proposed paradigms, the fog computing model [30] seems reasonable for locating the processing of tasks such as object recognition. This case has been revised in [31]; as a consequence, fog computation seems appropriate to be used as a service in some processes. The most recently developed processes involve delegating part of the computing to the edge. In [32], an architecture that works at the edge demonstrated efficiency in processing images close to the devices. Current architectures work mainly with fixed sensors in the city environment. In the case of our proposed architecture, the sensors are placed in vehicles. These vehicles may coincide in nearby spaces; in this case, the edge would be the best location for computing. However, considering the fact that the coincidence time is relatively short, we locate the process of object recognition in the edge and the process of integration in the fog. This location in the fog, with the possibility of moving to the edge, is one of the novelties of our proposed architecture. Current architectures offer connection-oriented communications systems to obtain information in a distributed manner. Another novelty of our proposed architecture is the use of a latency-aware publish–subscribe communications middleware: Middleware with latency awareness is required, as vehicles must have a high degree of certainty when making a decision. fog clustering is based on the topics of the publish–subscribe middleware. These last novel aspect is an interesting use of topics: When a vehicle is interested in an object type, it can use a common topic for that object. All vehicles interested in this type of object (e.g., a traffic cone) use the same topic. Our work differs from the existing works in the literature, as it considers a dynamic clustering of nodes at the fog level.

## 3. Proposed Architecture

**Figure 4:**
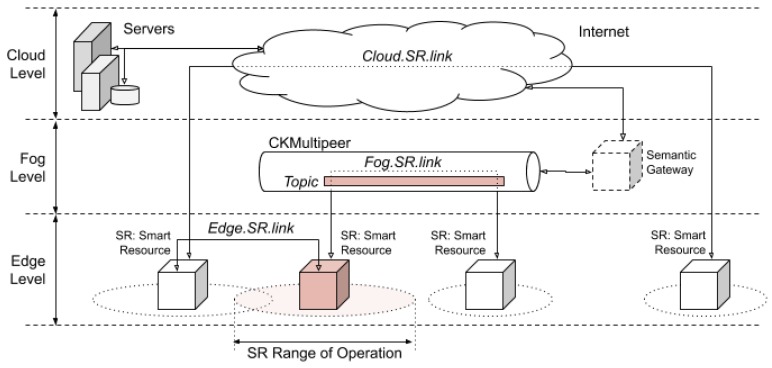
Location of the smart resources in the architecture. Edge interaction is possible when smart resources are physically in contact; that is, when their operating ranges overlap. Interaction in the fog allows communication with real-time restrictions between smart resources, without the need to share physical space. Cloud interaction allows connection to other components and data servers without real-time restrictions.

In this paper, according to the concepts of cloud, fog, and edge computing [8] and the requirement for elements capable of interacting at all layers, an architecture whose components are based on the smart resources has been designed (see Figure 4).

**Figure 5:**
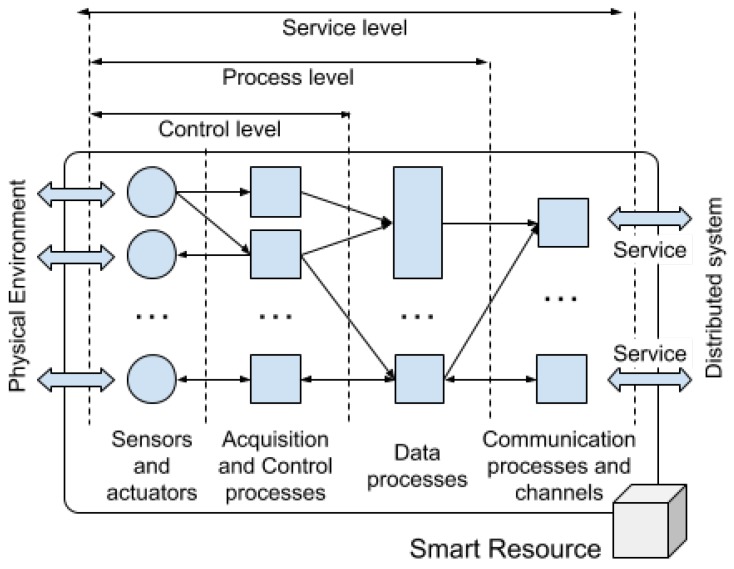
Concept and components of a smart resource. From interaction with the physical world (**left**) to interaction with the rest of the system **(right**).

Smart resources have been proposed, by the authors of this paper, in previous studies [33]. Smart resources (Figure 5) allow high connection flexibility, as the features are offered as services. The services offered depend on the available sensors and the computing capacity of each smart resource. Clients determine the necessary services, establishing a connection topology depending on their needs. For example, in the case of a smart resource that detects and identifies an object with a high probability, more information to corroborate the identified object may not be required; however, if the probability is low, the smart resource will need other measurements from other sensors that allow an increase in probability of successfully identifying the object.

A smart resource is defined as an element of intelligent control that offers capabilities for interaction with the environment through services. As an intelligent control element, it has a direct connection to the physical environment through a set of sensors and actuators. In order to carry out control actions, the smart resource has the functions of acquisition, reactive processing, and action. Up to this point, a smart resource does not differ from a control node. For example, a traffic light with a VGA camera, a set of relays to control the light, and an Arduino Microcontroller with a network connection constitute a control node. The role of the microcontroller is to acquire and transmit images, as well as to receive orders to turn the lights on or off. However, depending on the processing capacity of the smart resource and the functionalities it offers, there will be a set of processes with a higher level of intelligence. If the device mentioned above was provided with a more powerful microprocessor that allows, for example, the storage of historical data to infer the evolution of traffic or to detect the number of vehicles waiting, it would then be a smart resource. These advanced features are offered, through services, to other smart resources or system elements.

Communication between smart resources, independent of their location, is provided by a communications system called CKMultiPeer, which was proposed by the authors of this paper in [34]. CKMultiPeer is based on the data distribution service (DDS) model, which has been proposed for use in mixed-criticality distributed systems [35].

Integration of sensory information is produced along all levels of the architecture, providing a layer of sensory fusion to enrich the semantic meaning of the information provided to other components. The integration of sensory information is based on the enrichment of the semantic meaning of the information, depending on the architecture level at which it is integrated: The higher the architectural level, the more semantic meaning. For example, at a lower level of the architecture, all the values of a temperature sensor can be provided but, at a higher level, the information could be the average of these values and its comparison with the values of other temperature sensors. Sensory information is transparently shared by the components of the architecture from their location. The components may be close or located in the cloud.

CKMultiPeer allows connections between smart resources at both the cloud and fog levels, or even at the edge level. In this last case, smart resources are physically very close (direct contact at the edge) and the communication channels used are specific (such as Bluetooth) or by direct connection (such as I2C).

The importance of having mechanisms for semantic information conversion has been previously discussed. For example, when a large number of calculations are required to obtain a daily average over a historical archive of temperature samples or to predict a trend with a time horizon of one day. These semantic conversions require knowledge (for example, the temperature history found in the cloud) and some data found directly within the smart resources. The element that allows semantic conversions between the fog and the cloud is called the Semantic Gateway. The Semantic Gateway acts as a broker which can provide information to the edge.

**Figure 6:**
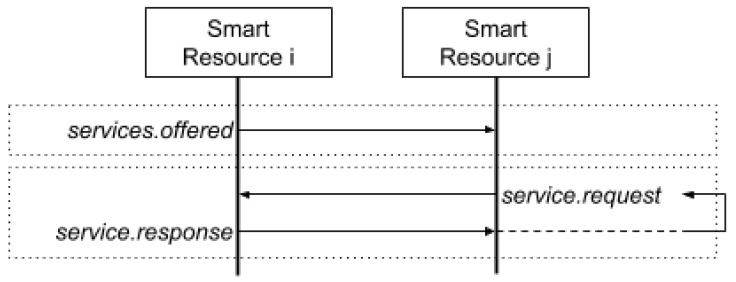
Communication protocol diagram between two smart resources at the edge level (edge.SR.link).

Connections in the edge are possible when two smart resources are in the same physical space or operating range. The operating range is defined as the physical space where the sensors and actuators of a smart resource can interact. For example, when a person rides a bicycle, the sensors of the bicycle can connect and collaborate with the sensors on the person; for example, the position sensors in the mobile device of a cyclist can collaborate with a camera installed on the bicycle to transmit the route or to recognise objects. This collaboration is interesting, because the same device can collaborate with other devices during different intervals of time. Therefore, a communication protocol that allows the collaboration between heterogeneous devices is necessary. Figure 6 presents the proposed service-oriented protocol.

The diagram in Figure 6 is located in the application level. When two smart resources have been connected, both offer their services by exchanging a JSON message. A smart resource i offers its services to another smart resource j. When smart resource j requires a service of smart resource i, it will request it and smart resource i will generate a response with the result or information provided by the service; this response may include an action, as well.

At the fog level, communication through CKMultipeer is done through topics. A topic is a common communication space in which information can be published. The element that publishes the information writes the data in a specific topic. The elements that wish to know the information of the topic subscribe to the topic and, when the information changes, they receive a message with the new data.

**Figure 7:**
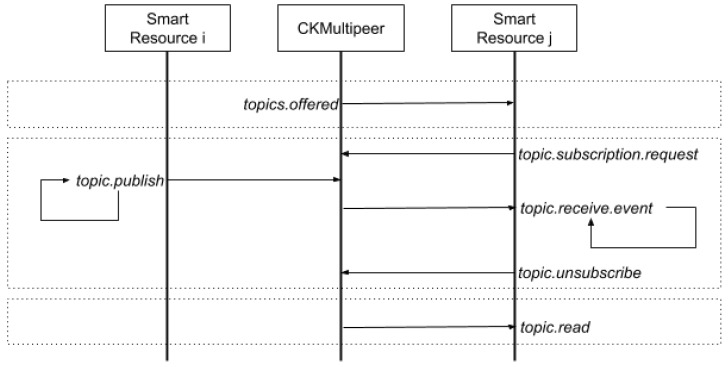
Communication protocol diagram between the CKMultipeer broker and smart resources at the fog level (fog.SR.link).

The communication protocol between two smart resources using CKMultipeer at the fog level is shown, in detail, in Figure 7.

The communication protocol at the fog level is based on the publish–subscribe paradigm. The exchange of services is carried out through topics and the smart resources interact in a decoupled manner. First, the smart resources request, at the fog level, the list of available Topics through the CKMultipeer broker. As a result, they receive a JSON file with the list of services offered, features, and service quality parameters [36]. When a smart resource requires a service, it subscribes to the associated topic through CKMultipeer. CKMultipeer is responsible for automatically sending all the information published in the topics to the relevant subscribers. When the smart resource which provides the required service (e.g., smart resource i in Figure 7) publishes new information in the associated topic, CKMultipeer notifies and sends the new information to all subscribed smart resources. Subscribers asynchronously read the information; that is, by means of a notification model based on events. When the smart resource does not need to receive more information, it can unsubscribe from the corresponding topic. Additionally, smart resources can read any topic without any subscription by means of a blocking operation.

## 4. Use-Case: Object Recognition

A use-case for object recognition was tested to validate the architecture. smart resources perform the functions of detecting objects using their own sensors, further enhancing the object certainty using information that they obtain from other smart sensors.

**Figure 8:**
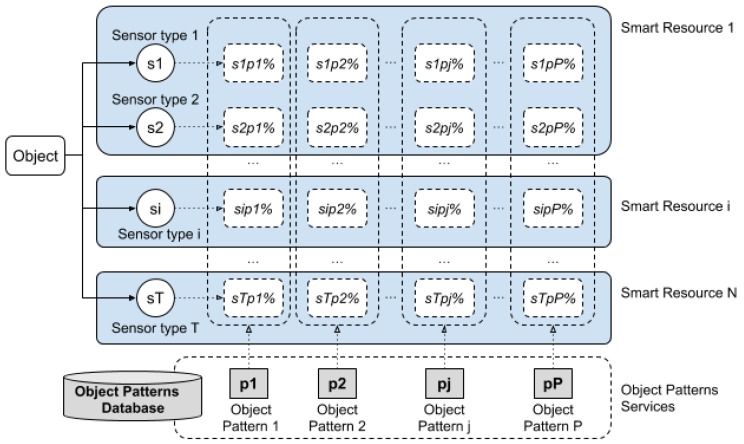
Smart resource scheme and connection with patterns for object recognition based on the analysis of the similarity between measurements and patterns.

As described in Section 2, the classification and integration steps imply the use of patterns, where the decision is based on the probabilities of each pattern provided from the different sensors. The process is described in Figure 8.

Taking into account that each object j has a specific pattern from each type of sensor i, a pattern is built with the object characteristics detected from a type of sensor. If the whole process is centralised, each device should have access to as many sensors as possible, as well as the patterns to compare with those sensors. However, the storage load of all the patterns and the processing load of all the sensors in the same device may be too much. In addition, in the case that a sensor obtains a very high probability with a specific object pattern, it is not necessary to continue processing data from more sensors, unless 100% certainty is required. Therefore, a distributed system can provide an adequate and efficient solution [37]. In this system, a device should request more results from other devices which have recognised an object only if it has a low certainty in the recognition of the object. Thus, a device only needs to have the patterns of the sensors used; moreover, it should be able to consult another device about an object, in order to reinforce the probability of the recognised object.

Smart resources offer, as services, their position in the map and the probability detected for each pattern, among others.

The communication system allows a device to connect to a source of information (e.g., the pattern of a specific object), from which it obtains data that can reinforce the identification of a specific object.

### 4.1. System Implemented

**Figure 9:**
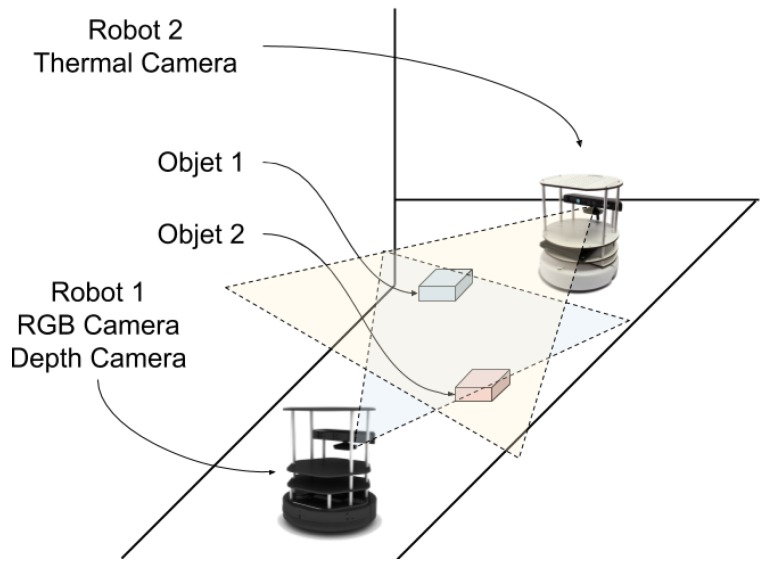
The case study used in the experiments. Vehicles are replaced by autonomous robots and street objects are replaced by boxes. The robots can be controlled better than a real bike or scooter, which allows the experiment to be replicated.

The implemented system is shown in Figure 9. Two Turtlebot [38,39] robots were used to carry the smart resources. Each smart resource was composed of one BeagleBone [40,41], the corresponding sensors, and one IEEE802.11 interface to allow communication between them. In the experiments performed, real-world vehicles were replaced by robots, where Turtlebot 1 carried Smart Resource 1 and Turtlebot 2 carried Smart Resource 2. Using well-known and controllable robots, the experiments can be replicated with vehicles having similar behaviour and movement.

**Figure 10:**
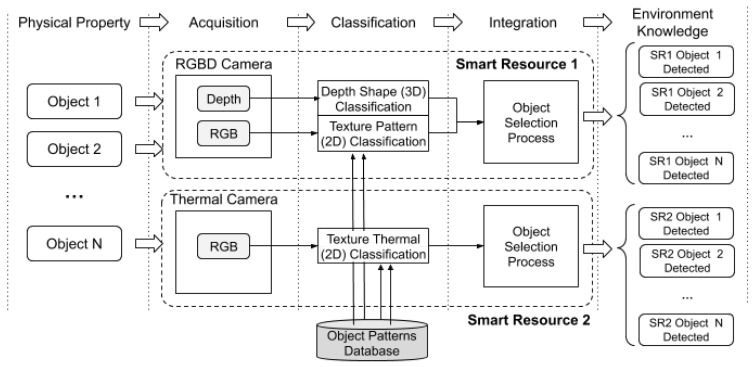
Details of the system implemented to perform the experiments and the corresponding step associated with the component (top of the figure).

Figure 10 shows the details of the implemented smart resources. The first smart resource used had two sensors, a depth camera [42] to detect the three-dimensional geometry, and an RGB camera to detect the two-dimensional texture. The second smart resource had only one sensor, a thermal camera that produced an RGB image associated to the colour reflected. The colour of the image depended on the temperature and was directly associated with the ink composition or box content.

The reason for using different RGB sensors (conventional vision and thermal vision, both 2D) was to be able to use the same recognition algorithms (2D image), but with different patterns of the same object. The acquisition step used a triple buffer [43] implementation which ensured that the sensor always had fresh data available without interfering with the classification process. The raw data classification process was based on the work presented in [44], which happens in three different steps: segmentation, blob detection, and feature recognition. First of all, the segmentation process allowed the device to extract the same colour and depth regions from the raw image. In the next step, some of these regions were grouped, forming image blobs using the seed region growing (SRG) technique [45]. Finally some shape, size, density, and colour characteristics were analysed to recognise some environment features, according to a set of available patterns, by means of a reliability-based particle filter. The integration was carried out by means of a Bayesian fusion algorithm with reinforcement associated with the different features provided by the different sensors. This algorithm has been described, in detail, in [46].

### 4.2. Experiment Performed

**Figure 11:**
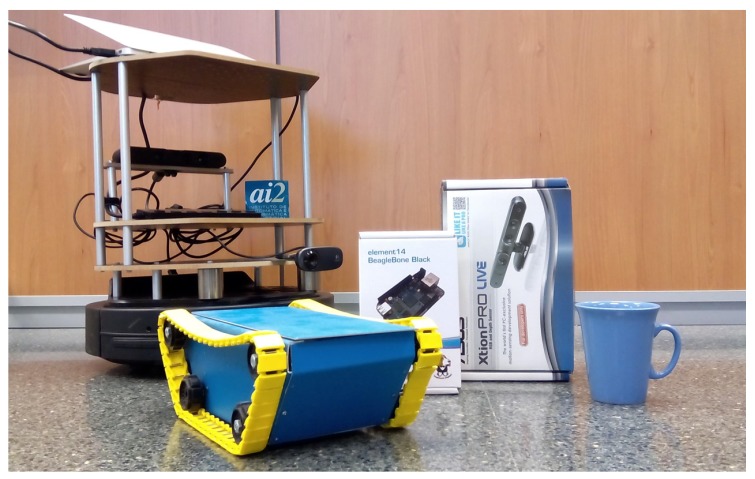
Objects (boxes) used in the experiments. Other objects are used to measure the false positive rate.

The objective of the experiment was to characterise the performance of the presented architecture. The experiments that were performed evaluated the obtained results in both single- and multi-robot approaches. While the single robot experiments offered information for characterising the access to on-board smart devices, the multi-robot approach demonstrated how to deal with spatially decoupled sensors. In order to provide these statistical values, a set of environmental features were recognised and integrated as environmental knowledge. This set included heterogeneous object samples (Figure 11), which were representative for testing.

Two specific objects (the boxes in the centre of Figure 11) were proposed for detection and recognition by means of the two different smart resources. Both objects had the same geometry (boxes) but different textures (the box of a Xtion and the box of a BeagleBone).

**Figure 12:**
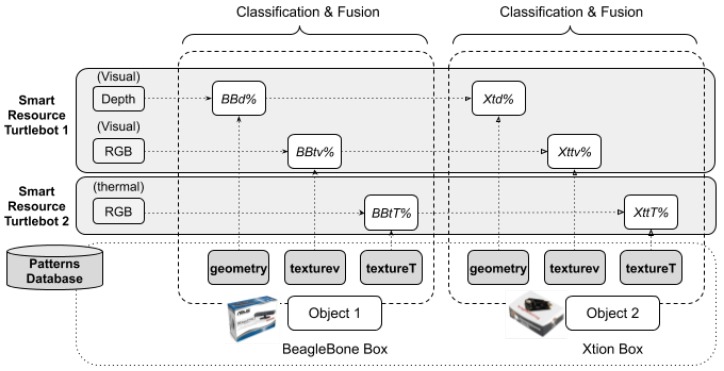
The objects (boxes) used in the experiments, sensors in the smart resources, and patterns used to recognise the boxes.

The patterns used to recognize the textures were the images of the six sides of each face of the boxes to be detected. Both smart resources contained both the texture (images) and geometry (3D shape) patterns of the two boxes. Therefore, the matching pattern sensor measurement was performed in both smart resources. Figure 12 shows the processes that were carried out based on smart resources and boxes.

**Figure 13:**
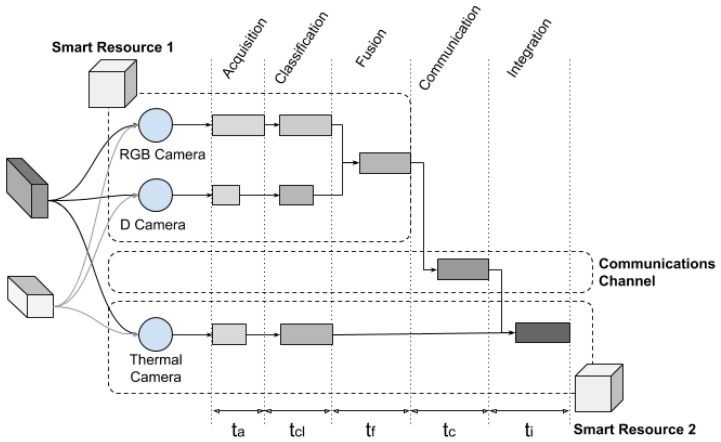
Data path of the experiment performed. From the data acquisition (**left**), by means of the smart resources sensors, to the result obtained from the integration of the information (**right**).

The experiment started when the two robots found the box. First, the robots were tested with the Beaglebone box; then, the Xtion box was used. When Smart Resource 1 detected a box with a reasonable prospect of certainty (greater than 0.5), it published the position of the estimated box, time, and certainly value in one of the topics ‘BBBox’ or ‘XtionBox’. A topic is a common space to share data in a publish–subscribe system. Smart Resource 2 then received the data of the certainty of both boxes and integrated the information with the data obtained from its sensors. The transmission time between both smart resources was also considered, as shown in Figure 13.

At the bottom of Figure 13, the times taken by each smart resource to classify an object are shown. The time $t_{a}$ refers to the acquisition time of the images by the cameras used. In the experiments, it remained constant depending on the sensor used.

The time $t_{c}l$ is the time it took to classify the images, according to the available patterns. In order not to alter the experiments, the patterns were already pre-loaded in each intelligent resource. When a pattern was not available, the smart resource had to request it, either from the fog through CKMultipeer or from the cloud.

The time $t_{f}$ is the time it took to fuse various results. An extended Kalman filter (EKF), which has been commonly used in similar environments, was used [47]. It is important that, once the object detected by the camera was classified, the percentage of certainty was used as the inverse error (i.e., higher certainty implies lower error) in the measurement of a sensor; that is, when comparing an image with a pattern, for example the BBBox box, it was considered to be a BBBox box by the sensor with a specific percentage of certainty. Integration may provide an increase in certainty in the recognition of an object but, for integration, the smart resources must communicate. The time $t_{c}$ is the communications time. A 54 Mbps WiFi network was used for CKMultipeer.

The time $t_{i}$ is the time it took to integrate the results. A summary of the average times obtained is shown in the results section.

## 5. Results

### 5.1. Latency Time

The average message latency times were obtained using the protocol presented in the previous section.

Figure 13 shows the total time taken to obtain the final result of information integration; that is, the addition of all times involved in the data path. Times were taken from processes running independently. Table 1 shows the estimated process times, based on the decoupled tasks and results. In the case that the smart resource had two sensors (turtlebot 1), the common times (e.g., $t_{a}$ or $t_{c}l$) considered were the worst of all sensor times.

It should be noted that the process times were similar, due to both smart resources using the same libraries, microcontroller boards, and the boxes to detect were quite similar. It can be seen that CKMultipeer introduced high latency. To check whether information integration was profitable, it is necessary to study if the increased latency time required to increase object recognition can justify the percentage of certainty improved. The ratio obtained using local integration and collaborative integration is studied in the next subsection.

### 5.2. Object Recognition Certainty

In the proposed scenario, the two robots (Turtlebot 1 and Turtlebot 2) navigated until they detected the same set of objects. Both robots had a different perspective and were located correctly on the map. To show the process better, the results of each detected object are shown separately.

Table 2 shows the results obtained when the data from the sensors of the first robot (Turtlebot 1) are compared with the geometries of the boxes. It can be seen that both were very similar, with a certain difference favourable to the BeagleBone box. In the case of texture, the RGB sensor had a clear tendency to detect the correct object.

As can be seen from Table 2, when Smart Resource 1 requested the system (through the CKMultiPeer topics) with the certainty of the object, the correct object was always reinforced. Furthermore, the data in the opposite direction, when the integration was done by Smart Resource 1 and the information of certainty was provided by Smart Resource 2, were similar. Consequently, it is possible that two uncoupled, heterogeneous systems can collaborate to improve their perception of objects.

Turtlebot 2 also detected the two objects, but only by means of texture. Consequently, the smart resource of the Turtlebot 2 requested the texture service to the system and, upon receiving the data, the correct object was reinforced. When merging the information, the object recognized as the BeagleBone box by Turtlebot 2 was reinforced much more than the Xtion box object (Table 3). In the case of the second object, the same trend can be observed, but it was the Xtion box that was reinforced.

However, in all cases, the certainty of the wrong object also increased. This was an expected behaviour, due to the algorithm used for the integration, which should not be a problem as the certainty of the incorrect object increased in a lesser proportion than the certainty of the correct object. However, the integration algorithm could be modified to correct the certainty, in such cases.

### 5.3. Information Integration

To understand the improvements that the fog brings, edge accuracy was compared with the accuracy obtained by information integration of the two smart resources (Table 4). In Table 4, the optimization value was obtained by dividing the best edge accuracy by the fog accuracy obtained from the information integration process. The cost of integration was obtained by dividing optimization by latency, which indicates how many milliseconds we needed to spend to increase the accuracy by one percent.

From the results presented above, it can be seen that the local processing of information was less expensive in time than that of information integration. On average, information integration spent six times the amount of time for classification and subsequent local integration. However, information integration provided an improvement in the certainty of object recognition. With the results obtained, the amount of additional time needed to improve a low percentage in recognition was high.

## 6. Conclusions

The proposed system allows a smart resource to decide whether the percentage of local certainty in recognizing an object is enough to make a decision, or if it is better to employ information integration using other smart resources to reinforce the recognition certainty. For example, if a user is looking for a specific object, such as a waste bin, it is probably enough to recognize it with low certainty and wait for the vehicle to recognize another with a higher percentage; after all, there are many waste bins in a city. However, if the object recognized with little certainty is a traffic cone, which indicates a problem or a potential accident, it is better for the vehicle to decrease their speed and ask nearby smart resources for more information and integrate it, in order to increase the certainty rate and decide between avoiding an obstacle or stopping the vehicle to avoid a collision.

The system used and experiments performed are placed in the edge and fog levels. The fog level implies the use of a common communication channel between all smart resources; this communication channel must manage the connections between smart resources and third parties. The use of a common communication channel provides the smart resources transparent and decoupled access to information. If two (or more) smart resources need to be coupled (for example, to share high-speed or high-frequency information), it is necessary to use a communication channel close to the edge level. This communications channel can be oriented to a physical connection, such as I2C, or wireless connection, such as Bluetooth.

In the presented use-case, the cloud was not considered. This was because this level manages large amounts of complex data and, consequently, involves higher computational times. The cloud is used, mostly, to store new patterns or update existing ones. As such, it can be used as a repository, allowing smart resources to have more detection power and locally adapt the patterns to different environments. The cloud allows smart resources to push the power limits of microprocessor computation and storage.

The combination of sensors, actuators, micro-controllers, and communications interfaces allows smart cities to be implemented by means of distributed intelligent systems. This paper proposes a general latency-aware architecture to integrate sensor information, which is focused on the results of increasing certainty in the recognition of objects. Due to of the large number and variety of sensors which exist in smart cities, it is convenient to organize them into devices that can interact with each other. Increased accuracy in object recognition is based on node collaboration by integrating information from nearby nodes. The experiments carried out to verify the integration of the information increased the certainty and, consequently, the success in object detection and recognition.

Based on the results, it is possible to apply information integration in smart cities as a method to improve the services offered by different elements. The proposed system, with two image processing devices, serves as a proof-of-concept case. The use-case had only two vehicles and integrated three sensors, which was useful to validate the idea but suggests that research in new scenarios can clarify the relationships between optimisation and latency. Constructing use-cases with more sensors and vehicles will allow a device to make decisions about whether waiting for the integration process from other devices is necessary to improve the optimisation, or if the device can work with self-assurance. The results obtained showed only a small increase in certainty. However, this does not detract from the validity of the proposed architecture. Different kinds of sensors and detection or fusion algorithms may obtain different results, which may present better outcomes, as they do not depend on the proposed architecture. Furthermore, in this work, we assumed that the devices could not reach each other directly without going through the fog layer (using the publish–subscribe paradigm), as our vehicles were in motion. However, as future work, this use-case could be interesting to investigate, as it could reduce the latency in other scenarios. For example, it is interesting to consider the case when the devices can reach each other directly without going through the fog layer. This use-case would be interesting to investigate with a dynamic layer placement of the devices (i.e., from fog to edge and vice versa), which may be useful in deciding the best layer to use, depending on the desired certainty increase or the latency decrease.

## Figures and Tables

**Table 1:** Results of the average estimated times in each stage of the process. The acronym “n.a.” (not appropriate) means that time is not provided because that phase was not done in the experiment.

Object	SR	$t_{a}$	$t_{c}l$	$t_{f}$	$t_{c}$	$t_{i}$	Total
BBox	turtlebot 1	16 ms.	12 ms.	25 ms.	n.a.	n.a.	53 ms.
	turtlebot 2	32 ms.	18 ms.	n.a.	n.a.	n.a.	50 ms.
	Integration (fog)	n.a.	n.a.	n.a.	244 ms.	23 ms	320 ms.
XtionBox	turtlebot 1	16 ms.	15 ms.	24 ms.	n.a.	n.a.	55 ms.
	turtlebot 2	32 ms.	17 ms.	n.a.	n.a.	n.a.	49 ms.
	Integration (fog)	n.a.	n.a.	n.a.	244 ms.	25 ms.	318 ms.

**Table 2:** Results of certainty for correct object detection applying the integration method with two similar objects (BeagleBone box).

Object: BeagleBone Box	Turtlebot 1			Turtlebot 2	
**Object Pattern**	**Geometry**	**Texture**	**Fusion**	**Texture**	**Integration**
BeagleBone box	0.726	0.671	0.792	0.789	0.824
Asus Xtion box	0.647	0.127	0.651	0.192	0.658

**Table 3:** Results of certainty for correct object detection applying the integration method with two similar objects (XTion box).

Object: Xtion Box	Turtlebot 1			Turtlebot 2	
**Object Pattern**	**Geometry**	**Texture**	**Fusion**	**Texture**	**Integration**
BeagleBone box	0.243	0.231	0.253	0.210	0.259
Asus Xtion box	0.851	0.712	0.886	0.812	0.902

**Table 4:** Comparison results between edge accuracy and fog accuracy integration (only done by Smart Resource 2).

		Local (Smart Resource)		Fog		Integration	
**Object**	**SR**	**Accuracy**	**Latency**	**Accuracy**	**Latency**	**Optimization**	**Integration Cost**
BBBox	1	0.792	53 ms.				
	2	0.789	50 ms.	0.824	320 ms.	4%	79.2 ms
xTionbox	1	0.886	55 ms.				
	2	0.812	49 ms.	0.902	318 ms.	2%	176.1 ms.

## Abbreviations

The following abbreviations are used in this manuscript:

DDS	Data Distribution Service
IoT	Internet of Things
RAMI 4.0	Reference Architectural Model Industrie 4.0
RGB	Red, Green, Blue
SR	smart resource
